# Identification of hypermethylated CpG sites mapped to *LIFR* as specific diagnostic biomarkers of colon cancer

**DOI:** 10.1016/j.gendis.2024.101334

**Published:** 2024-05-19

**Authors:** Ruizhi Chang, Ganxun Li, Guan-nan Jin, Bixiang Zhang, Ze-yang Ding

**Affiliations:** Department of Surgery and Hubei Key Laboratory of Hepatic-Pancreatic-Biliary Diseases, National Medical Center for Major Public Health Events, Tongji Hospital, Tongji Medical College, Huazhong University of Science and Technology, Wuhan, Hubei 430030, China

Despite the improvement of programmatic screening, adjuvant chemotherapy, and curative resection, colon cancer, with increasing incidence and mortality rates, still poses a significant public health challenge as the third most commonly diagnosed and second most fatal cancer worldwide.^1^ The myriad of benefits, encompassing the improved survival rates, treatment outcomes, and cost efficiency, further underscore the criticality of early colon cancer diagnosis. Hence, there is a pressing need to identify more potent biomarkers for improving the accuracy of colon cancer diagnosis. In summary, we identified three methylation-related biomarkers that exhibit excellent diagnostic performance, suggesting their significant potential for its application as noninvasive clinical biomarkers in the diagnosis of colon cancer.

To identify sensitive and specific methylation biomarkers for colon cancer, we organized a rigorous workflow to detect methylation biomarkers by comparing colon cancer patients with normal controls and patients with other tumors (Fig. 1A). Starting from 3374 hypermethylated CpG sites located in 2171 differentially expressed genes in colon cancer, we removed 1373 sites with mean methylation levels higher than 0.1 in 38 normal tissues from the Cancer Genome Atlas (TCGA), 43 normal colon tissues (GSE66555), and 184 blood samples from healthy individuals (GSE69270). The methylation profiles of these 2001 CpG sites across distinct datasets were graphically represented, demonstrating a clear demarcation between tumor samples and normal controls, underscoring the robustness of our findings (Fig. 1B). Finally, after further filtering these 2001 CpG sites, we identified three colon cancer-specific CpG sites that were hypermethylated only in patients with colon cancer and not in patients with 28 other tumors from the TCGA (Fig. 1C).

**Figure 1:**
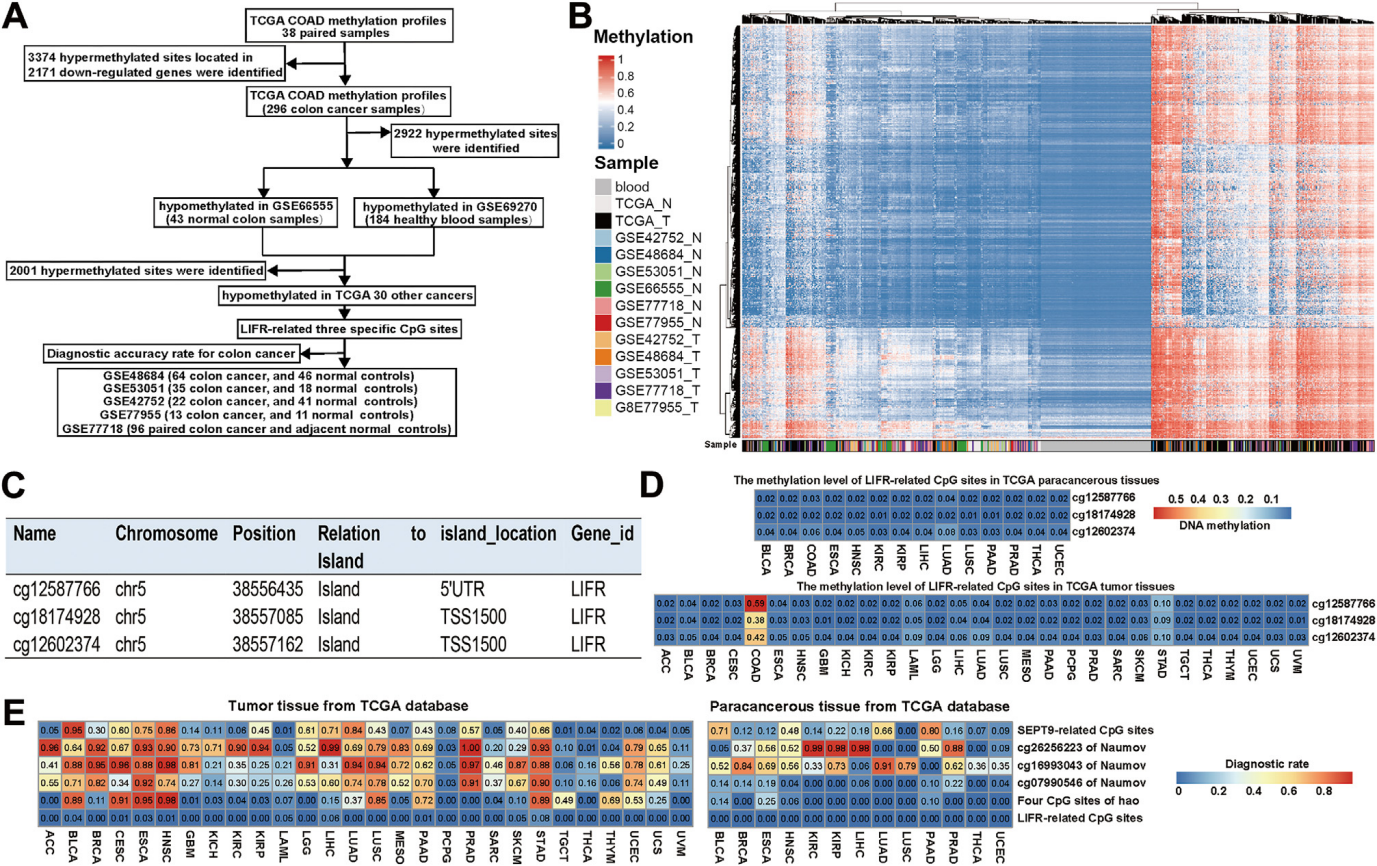
Identification of colon cancer-specific hypermethylated sites. **(A)** Protocol for finding candidate diagnostic biomarkers for colon cancer. **(B)** Unsupervised hierarchical clustering of colon cancer patients and normal controls using 2001 hypermethylated CpG sites located in hypomethylated genes. The heatmap shows the methylation levels of 2001 CpG sites in eight datasets (TCGA, GSE69270, GSE66555, GSE48684, GSE53051, GSE42752, GSE77718, and GSE77955). Normal controls were clustered together and separated from colon cancer patients. **(C)** The average methylation level of three colon cancer-specific CpG sites in colon cancer and 28 other cancers. **(D)** Detailed information on three hypermethylated CpG sites mapped to *LIFR*. **(E)** Comparison of the methylation markers explored in this study with methylation markers published previously. The rows show different sources of methylation biomarkers. The horizontal axis shows the different methylation datasets. The upper panel indicates the proportion of specific sites diagnosing normal tissue as colon cancer, and the lower panel indicates the proportion of specific sites diagnosing tumor tissue as colon cancer. ACC: adrenocortical carcinoma; BLCA: bladder urothelial carcinoma; BRCA: breast invasive carcinoma; CESC: cervical squamous cell carcinoma and endocervical adenocarcinoma; ESCA: esophageal carcinoma; HNSC: head and neck squamous cell carcinoma; GBM: glioblastoma; KICH: Kidney chromophobe; KIRC: kidney renal clear cell carcinoma; KIRP: kidney renal papillary cell carcinoma; LAML: acute myeloid leukemia; LGG: brain lower grade glioma; LIHC: liver hepatocellular carcinoma; LUAD: lung adenocarcinoma; LUSC: lung squamous cell carcinoma; MESO: mesothelioma; PAAD: pancreatic adenocarcinoma; PCPG: pheochromocytoma and paraganglioma; PRAD: prostate adenocarcinoma; SARC: sarcoma; SKCM: skin cutaneous melanoma; STAD: stomach adenocarcinoma; TGCT: testicular germ cell tumors; THCA: thyroid carcinoma; THYM: thymoma; UCEC: uterine corpus endometrial carcinoma; UCS: uterine carcinosarcoma; UVM: uveal melanoma.

The three CpG sites specific to colon cancer were found to be uniquely associated with the leukemia inhibitory factor receptor (LIFR) gene. Given the general co-methylation tendency of CpG sites in nearby regions, the correlation between these specific biomarkers and other CpG sites located within 2000 base pairs was examined (Fig. S7). Notably, the CpG sites in the neighboring regions exhibited a strong positive correlation with the three colon cancer-specific CpG sites, consistent with prior findings.^2^ Analysis of the methylation levels across different patient subgroups revealed that these three CpG sites were significantly hypermethylated in the CpG Island Methylator Phenotype-High (CIMP-H) subgroup and hypomethylated in the CpG Island Methylator Phenotype-Low (CIMP-L) subgroup (Fig. S8). Moreover, even in stage I patients, two out of the three specific CpG sites demonstrated significant hypermethylation (Fig. S9). Consequently, these identified CpG sites are promising biomarkers for the diagnosis of colon cancer.

The methylation profiles of 38 paired colon cancer and normal control samples from the TCGA were utilized as a training cohort. Additionally, five independent datasets (GSE48684, GSE53051, GSE42752, GSE77718, and GSE77955) were used as validation cohorts comprising 230 colon cancer samples and 212 normal samples. We developed a logistic regression model to predict colon cancer based on a combination of three CpG sites mapped to LIFR, as well as the entirety of the CpG sites. By employing a combination of three specific CpG sites (cg12587766, cg184928, and cg12602374) mapped to LIFR, we achieved a receiver operating characteristic area greater than 0.93 in the majority of the validation cohorts (Table S7). Given the shared characteristics between colon cancer and rectal cancer, such as the anatomical continuity, common tissue origin, treatment strategies, and multi-omics features, we assessed the diagnostic efficacy of our specific biomarker sites in rectal cancer. The results revealed the strong performance of LIFR-related hypermethylated sites as diagnostic biomarkers for rectal cancer in an independent rectal cancer (TCGA-READ) cohort (Fig. S10). Overall, a combination of three specific CpG sites has shown promising potential as a diagnostic biomarker for the context of colorectal cancer.

We systematically compared our diagnostic model with methylation biomarkers for colon cancer published in prior academic literature. Logistic regression models were developed using various feature sets, including three CpG sites from our study, four from Hao et al,^3^ five from SEPT9 in colon cancer samples, and three distinct CpG sites from Naumov et al.^4^ The precision and sensitivity in distinguishing colon cancer patients from normal controls were significantly different and consistent with the different datasets (Table S7). Additionally, we investigated the efficacy of various methylation biomarkers in discriminating colon cancer from other malignancies. Notably, tumor and normal samples from 28 different tumors were rarely predicted to be colon cancer, with a range of 0% to 7% and a median of 0.59%, based on our specific CpG sites for colon cancer (Fig. 1D). However, the predictions based on the CpG sites of Hao et al and Naumov et al as feature sets varied from 0% to 100%, with medians of 60.34% and 26.23%, respectively (Fig. 1E). Taken together, these results suggested that the combination of three specific CpG sites could be used to stratify colon cancer patients effectively from other cancer types, suggesting the potential utility of these sites in determining the tissue origin of cancers of unknown primary origin (CUPs).

One significant finding of this study was the identification of several colon cancer-specific CpG sites that could serve as potential diagnostic biomarkers for colon cancer. An additional clinical implication of our findings is their potential utility in ascertaining the tissue origin of CUPs, which constitute 3%–5% of all cancer diagnoses. Immunohistochemical algorithms can be instrumental in accurately identifying the primary site in several CUP patients. For instance, a previous study delineated a CUP characterized by CDX-2, CK20 and CK-7 staining profiles, designating it as the colon cancer profile CUP (CCP-CUP).^5^ Furthermore, identification of the primary site in CUPs by sequencing has been ongoing, and our findings may provide evidence for identifying the primary site of colon cancer in patients with CUPs through DNA methylation analyses.

Although our study offers proof of concept regarding the potential of utilizing large-scale genome-wide methylation data from diverse cancer types to investigate tissue-specific methylation for colon cancer diagnosis, there are still several limitations. First and foremost, our endeavors were based on a retrospective design, highlighting the need for prospective studies to validate our findings. Moreover, in this study, we also found that three methylated sites mapped to LIFR also exhibited good performance in diagnosing rectal cancer; however, only one rectal cancer cohort was included in this study, and a validation cohort of rectal cancer patients was lacking to confirm these findings. Furthermore, to improve the clinical application of LIFR, it is imperative to assess its biological functions and molecular mechanisms in the context of colorectal cancer. Finally, while our markers exhibited hypermethylation in tissue samples from colon cancer patients, the potential of these markers to serve as noninvasive biomarkers for colon cancer requires further investigation.

In summary, we identified three methylation-related biomarkers that exhibit excellent diagnostic performance, suggesting their significant potential for its application as noninvasive clinical biomarkers in the diagnosis of colon cancer.

## Conflict of interests

ZD served as a speaker and consultant for Bayer, Eisai, Roche, MSD, Astra-Zeneca, Innovent, Hengrui, and BeiGene. The remaining authors declare that they have no competing conflict of interests.

## Funding

This work was supported by the National Nature Science Foundation of China (No. 82273441, 81874065), the National Basic Research Program of China (No. 2020YFA0710700), the Knowledge Innovation Program of Wuhan-Shuguang Project (China) (No. 2022020801020456), Tongji Hospital (HUST) Foundation for Excellent Young Scientist (China) (No. 2020YQ05), and the First Level of the Public Health Youth Top Talent Project of Hubei Province (China) (No. 2022SCZ051).

## Data availability

The data that support the findings of this study are available from the corresponding author upon reasonable request.
