# Supplementary material for: Identification of hypermethylated CpG sites mapped to LIFR as specific diagnostic biomarkers of colon cancer
Source: Genes Dis. 2024 May 19;12(2):101334. doi: 10.1016/j.gendis.2024.101334 (PMC11616024; doi:10.1016/j.gendis.2024.101334)
Supplement: Multimedia component 1 [file mmc1.docx]

**SUPPORTING DATA**

#### Identification of hypermethylated CpG sites mapped to *LIFR* as specific diagnostic biomarkers of colon cancer

Ruizhi Chang^1, #^, Ganxun Li^1, #^, Guan-nan Jin^1,2^*, Bixiang Zhang^1,^*, Ze-yang Ding^1,^*

^1^Department of Surgery, and Hubei Key Laboratory of Hepatic-Pancreatic-Biliary Diseases, National Medical Center for Major Public Health Events, Tongji Hospital, Tongji Medical College, Huazhong University of Science and Technology, Wuhan, China

^2^Present address: Department of Internal Medicine, Union Hospital, Tongji Medical College, Huazhong University of Science and Technology, Wuhan, China

^#^These authors contribute equally to this work.

***Correspondence to:** Ze-yang Ding, M. D., Bixiang Zhang, M.D., Ph.D.; or Guan-nan Jin M. D.; **E-mail:** zyding@tjh.tjmu.edu.cn; [bixiangzhang@hust.edu.cn](mailto:bixiangzhang@hust.edu.cn); or jgnlh@163.com

**Table of Contents**

**Supporting Methods**

Data acquisition (Page 4)

CpG island methylator phenotype (Page 5)

Mutation analysis (Page 6)

Identification of CIMP–immune cell associations (Page 6)
Differential analysis of DNA methylation and gene expression (Page 6)

Correlation between DNA methylation and gene expression (Page 7)

Identification of candidate diagnostic biomarkers (Page 8)

Evaluation of candidate diagnostic biomarkers (Page 8)

Statistical analysis (Page 8)

**Supporting Results** (Page 10)

**Supporting Discussion** (Page 14)

**References** (Page 15)

**Supporting Figures**

**Figure S1.** The DNA methylation landscape of colon cancer (Page 18).

**Figure S2.** Three methylation clusters were identified based on the k-means consensus clustering in GSE42752 colon cancer dataset (Page 20).

**Figure S3.** The DNA methylation landscape of colorectal cancer (Page 21).

**Figure S4**. Comparison of the expression of check points in the three methylation clusters (Page 22).

**Figure S5.** Volcano plot of differential methylation CpG sites and expression genes (Page 23).

**Figure S6** Relationship between DNA methylation and gene expression in colon cancer (Page 24).

**Figure S7** co-methylated between other CpG sites in neighboring regions of three specific biomarkers. The coefficients were calculated by Pearson correlation analysis (Page 25).

**Figure S8** CIMP related methylation of three colon cancer-specific CpG sites (Page 26).

**Figure S9** Stage related methylation of three colon cancer-specific CpG sites (Page 27).

**Figure S10** Performance of LIFR-related hypermethylated sites as diagnostic biomarkers of rectal cancer (TCGA-READ cohort) in TCGA datasets (Page 28).

**Supporting Tables**

**Table S1** Methylation information for 171 most variable CpG sites mapped to TSGs in the TCGA dataset (Page 29).

**Table S2** Characteristics of patients generated from TCGA in three methylation subgroups (Page 34).

**Table S3** Distribution of significantly mutated genes in three methylation subgroups (Page 35).

**Table S4** Distribution of differentially methylated CpG sites according to the distance to the TSS and CpG island (Page 36).

**Table S5** Genome-wide cis-regulation between DNA methylation and gene expression according to the distance to the TSS (Page 37).

**Table S6** Genome-wide cis-regulation between DNA methylation and gene expression according to the distance to the CpG island (Page 38).

**Table S7** Comparison of the performance of different methylation markers for classifying colon cancer and normal tissues (Page 39).

**Supporting Methods**

**Data acquisition**

Genomic, transcriptomic, and clinical profiles of colon cancer were sourced from The Cancer Genome Atlas (TCGA) projects [https://portal.gdc.cancer.gov/]. Level three DNA methylation profiles (Illumina HumanMethylation450 BeadChip), including 296 colon cancer and 38 normal cases, were acquired from the TCGA by a Bioconductor package *TCGA-Assembler 2* (1), and the genomic annotation of the CpG sites were based on the Bioconductor package *IlluminaHumanMethylation450kanno.ilmn12.hg19*. For each CpG site, there are two measurements: a methylated intensity (denoted by M) and an unmethylated intensity (denoted by U). The methylation status of each CpG was commonly expressed as beta-values (β = M/(M+U), ranging from 0 to 1) (2). The level three Gene expression profiles with log2 transformed normalized count were also obtained using *TCGA-Assembler 2* (1), which included 296 colon cancer and 38 normal controls. Then, curated somatic mutation profiles were downloaded through the *TCGABiolinks* Bioconductor package (3), including 291 patients with colon cancer. In addition, the methylation profiles for another 28 types of tumors with sample size more than 50 were collected from TCGA portal: ACC (Adrenocortical carcinoma, 80 tumor, 0 normal), BLCA (Bladder urothelial carcinoma, 419 tumor, 21 normal), BRCA (Breast invasive carcinoma, 798 tumor, 97 normal), CESC (Cervical squamous cell carcinoma and endocervical adenocarcinoma, 309 tumor, 3 normal), ESCA (Esophageal carcinoma, 186 tumor, 16 normal), HNSC (Head and Neck squamous cell carcinoma, 530 tumor, 50 normal), GBM (Glioblastoma, 153 tumor, 0 normal), KICH (Kidney Chromophobe, 66 tumor, 0 normal), KIRC (Kidney renal clear cell carcinoma, 325 tumor, 160 normal), KIRP (Kidney renal papillary cell carcinoma, 276 tumor, 45 normal), LAML (Acute Myeloid Leukemia, 194 tumor, 0 normal), LGG (Brain Lower Grade Glioma, 534 tumor, 0 normal), LIHC (Liver hepatocellular carcinoma, 380 tumor, 50 normal), LUAD (Lung adenocarcinoma, 475 tumor, 32 normal), LUSC (Lung squamous cell carcinoma, 370 tumor, 42 normal), MESO (Mesothelioma, 87 tumor, 0 normal), PAAD (Pancreatic adenocarcinoma, 185 tumor, 10 normal), PCPG (Pheochromocytoma and Paraganglioma, 184 tumor, 0 normal), PRAD (Prostate adenocarcinoma, 503 tumor, 50 normal), SARC (Sarcoma, 265 tumor, 0 normal), SKCM (Skin Cutaneous Melanoma, 473 tumor, 0 normal), STAD (Stomach adenocarcinoma, 395 tumor, 0 normal), TGCT (Testicular germ cell tumors, 156 tumor, 0 normal), THCA (Thyroid carcinoma, 515 tumor, 56 normal), THYM (Thymoma, 124 tumor, 0 normal), UCEC (Uterine corpus endometrial carcinoma, 439 tumor, 46 normal), UCS (Uterine carcinosarcoma, 57 tumor, 0 normal), and UVM (Uveal melanoma 80 tumor, 0 normal).

Moreover, seven DNA methylation datasets were downloaded from the Gene Expression Omnibus (GEO) database: GSE66555 (4) (43 normal colon samples), GSE69270 (5) (blood of 184 young Finns), GSE48684 (6) (64 colon cancer, 46 normal), GSE53051 (7) (35 colon cancer, 18 normal), GSE42752 (8) (22 colon cancer, 41 normal), GSE77718 (9) (96 paired colon cancer and adjacent normal) and GSE77955 (10) (13 colon cancer, 11 normal). The array platform of all datasets obtained from GEO was the HumanMethylation450 BeadChip (GPL13534). The CpG sites were all annotated by *IlluminaHumanMethylation450kanno.ilmn12.hg19*. Because all the data obtained from TCGA site and GEO database are deidentifed and displayed in public domain, the approval of Institutional Review Board (IRB) was waived.

**CpG island methylator phenotype**

A set of 535 human tumor suppressor genes (TSGs), down-regulated in colon adenocarcinoma samples relative to normal tissue samples from TCGA, was retrieved from the TSGene 2.0 database (11). To assess the CIMP phenomenon in colon cancer, CpG sites situated in the promoter regions of these TSGs—with a high standard deviation (SD > 0.2) of beta-values in 296 colon cancer tissues and low beta-values (mean β < 0.05) in 38 normal tissues—were chosen for further clustering analysis. This approach was consistent with findings from prior studies (12, 13). The *ConsensusClusterPlus* Bioconductor package was employed to execute consensus cluster analysis using the K-means algorithm (14). Correlations between clinical features and each cluster were determined using Fisher’s exact test. The source codes to identify the CIMP phenotype are available on the GitHub repository: <https://github.com/wolfgangsk07/CIMP_colon_cancer>. Dimensionality reduction analysis was conducted to screen the most important CpG sites using the *randomForestSRC* function from the R *randomForest* package (15). Based on the DNA methylation levels of these significant CpG sites, a multinomial log-linear model was constructed with the multinom function from the R *nnet* package to determine the CIMP status of colon cancer in the TCGA database.

**Mutation analysis**

Mutation data obtained from whole-exome sequencing were analyzed using the *maftools* package (16). Samples exhibiting missense mutations, nonsense mutations, multiple hits, splice-site mutations, frameshift insertions, frameshift deletions, in-frame insertions, or in-frame deletions were considered mutation-positive. Tumor mutational burden (TMB), an emergent biomarker for immunotherapy responses, is defined as the number of somatic, coding, base substitution, and indel mutations per megabase of a specified genome. The size of the human whole exome is approximated to be 35 Mb, based on the GRCh38 reference. To calculate the TMB per megabase, the cumulative count of mutations is divided by the size of the coding region of the given genome (17). Differences in mean values between pairs of subgroups were evaluated using the Mann–Whitney test, while the Kruskal-Wallis test was used for comparisons across three subgroups. Correlations between mutations in specific genes and each cluster were determined using Fisher’s exact test.

**Identification of CIMP–immune cell associations**

The associations between the CIMP status and immune cell abundance estimated by TIMER (18), XCELL (19), EPIC (20), and CIBERSORT (21) were evaluated by Fisher’s exact test with *P* less than 0.05 regarded as statistical significance.

**Differential analysis of DNA methylation and gene expression**

Among the 296 patients from the TCGA dataset, 38 had both colon cancer and corresponding normal methylation profiles. These paired colon cancer and normal methylation profiles were utilized for conducting differential methylation analysis. CpG sites with more than 20% missing values (designated as 'NA') were excluded, and the remaining 'NA' values were imputed using the k-nearest neighbor algorithm implemented through the Bioconductor package *'impute*.' Subsequently, the raw methylation data underwent preprocessing and normalization using the Bioconductor package *'minfi'* (22). To ensure data quality, low-quality methylation probes were filtered out based on the following criteria: I) presence of single-nucleotide polymorphisms (SNPs) within the assayed CpG dinucleotide (23); II) failure to uniquely map to the human reference genome (hg19) (24); III) localization on the sex chromosomes (25). Furthermore, differential methylation at CpG sites between the 38 tumors and their matched normal tissue samples was determined using a paired t-test. The False-discovery rate (FDR) based on the Benjamini-Hochberg method was used to calculate the adjusted P-value for each CpG site. Differentially methylated CpG sites with an adjusted P-value less than 0.05 and an absolute difference in beta-value greater than 0.2 were selected for further analysis.

Among the 38 patients obtained from the TCGA dataset, 26 patients had both colon cancer and corresponding normal expression profiles. These paired expression profiles were utilized for conducting differential expression analysis. Differentially expressed genes, based on raw read counts, were identified using the Bioconductor package *DESeq2* (26). Differentially expressed genes with an adjusted P-value less than 0.01 and an absolute log2 fold change greater than 1 were selected for subsequent analysis.

**Correlation between DNA methylation and gene expression**

Correlation analysis was conducted on a dataset comprising 277 tumor samples, each of which had both methylation and corresponding gene expression profiles. First, we examined cis-regulation, assessing the relationship between DNA methylation and the expression of associated genes by calculating Pearson correlation coefficients for each CpG site's methylation value and expression level. Subsequently, we explored trans-regulation, focusing on the association between one gene's methylation and the expression of another gene, employing Pearson correlation analysis. Trans-regulation analysis was restricted to differentially expressed genes, and the corresponding promoter CpG sites were selected from genes that exhibited simultaneous differential methylation and differential expression. We identified instances of a positive correlation, characterized by a correlation coefficient exceeding 0.3 and an adjusted P-value below 0.05, as well as negative correlations with correlation coefficients less than -0.3 and adjusted P-values less than 0.05.

**Identification of candidate diagnostic biomarkers**

Potential CpG sites serving as diagnostic biomarkers for colon cancer were identified in TCGA datasets through a systematic screening process. Initially, a comparative analysis of 38 paired colon cancer and normal tissues revealed hypermethylated CpG sites within low-expression genes specific to colon cancer. Subsequently, 296 colon cancer samples were juxtaposed with 38 normal tissues from TCGA, 43 normal colon tissues (GSE66555), and 184 blood samples from healthy individuals (GSE69270). CpG sites lacking significant hypermethylation differences were excluded from further analysis. In the next step, colon cancer-specific hypermethylated CpG sites were discerned by excluding CpG sites with mean methylation values exceeding 0.1 in tumor or normal samples obtained from 30 other cancer types available in the TCGA database (Table S1). Finally, candidate diagnostic biomarkers for colon cancer were meticulously selected based on the remaining CpG sites.

**Evaluation of candidate diagnostic biomarkers**

The colon cancer dataset originated from TCGA was utilized as the training cohort, while five other independent profiles (GSE48684, GSE53051, GSE42752, GSE77718, GSE77955) were used as validation cohorts. Based on the candidate diagnostic CpG sites, a logistic regression model was constructed to predict whether the unknown sample is a tumor or not. The accuracy of the predicted model was assessed based on the calculation of sensitivity and specificity.

**Statistical analysis**

Fisher’s exact test, Mann–Whitney test, Kruskal-Wallis test, paired t-test, Pearson correlation analysis, multinomial log-linear model, and logistic regression model were applied in this study as described above. All bioinformatic and statistical analyses were performed using the R software (version 4.1.0, and all *P* values were two-tailed. *P* value less than 0.05 was regarded as significant difference.

**Supporting Results**

**Genome-wide methylation landscape of colon cancer**

Based on 535 human tumor suppressor genes (TSGs) associated with colon cancer from the TSGene 2.0 database, along with DNA methylation profiles comprising 296 colon cancer samples and 38 normal controls sourced from the TCGA, we identified 171 of the most variably methylated CpG sites for unsupervised consensus cluster analysis **(Table S1)**. Patients were subsequently categorized into three distinct methylation clusters: the non-CIMP (cluster 1), CIMP-L (cluster 2) and CIMP-H (cluster 3) **subgroups (Figure S1)**. Cluster 3 (comprising 18.9%) exhibited widespread hypermethylation of CpG sites associated with TSGs, was primarily located in the promoter regions and was designated the CpG island methylator phenotype-high (CIMP-H) subgroup.

To assess the robustness of our method for identifying the CIMP phenotype, we validated the method in an independent cohort (GSE42752), and the results provided further support for our conclusions **(Figure S2)**. Upon investigating the relationship between the CIMP-H subgroup and clinical characteristics, we observed a significant correlation between the CIMP-H subgroup and both age and right-sided tumor location **(Table S2)**. Recognizing the similarities between colon cancer and rectal cancer, we conducted analogous cluster analysis on rectal cancer samples. The results indicated significant enrichment of CIMP-L in the distal colon and rectum, consistent with findings from previous studies **(Figure S3)**.

To demonstrate the clinical translation of our CIMP subtype findings, we employed a multinomial log-linear regression with a 70%/30% training/test set split, balanced class weights, and 5-fold cross-validation. A set of four probes (cg12690148, cg12828819, cg15778437, and cg05598562) that could accurately distinguish between CIMP and non-CIMP statuses with near-perfect accuracy was identified. The predictive accuracy reached as high as 0.887 in the TCGA cohort, suggesting the potential to significantly reduce economic costs and greatly enhance clinical utility.

Subsequently, we investigated whether the CIMP-H subgroup exhibited a significant correlation with a high frequency of specific somatic mutations using Fisher's exact test. Consistent with the findings of previous studies, tumors within the CIMP-H subgroup were distinguished by a significantly elevated frequency of BRAF mutations. In addition, we observed enrichment of SETD1B, NCOR2, and KMT2B mutations in the CIMP-H subgroup **(Table S3)**. Additionally, a notable disparity in mutational burden was observed among patients in different subgroups **(Figure S1B)**, with the CIMP-H subgroup exhibiting the highest TMB **(Figure S1C)**. Furthermore, by conducting propensity score matching analysis to account for confounding factors, including age, disease stage, and sex, we found that a higher frequency of TMB in the CIMP-H subgroup was independently correlated with global hypermethylation of TSGs in the promoter region. Hence, the increased frequency of TMB in the CIMP-H subgroup likely corresponds to global hypermethylation of TSGs in the promoter region. Additionally, we detected increased infiltration of immune cells, including CD4+ and CD8+ T cells, B cells, and NK cells, in the CIMP-H cohort, which was indicative of enhanced immunogenicity **(Figure S1D)**. Moreover, the checkpoint genes exhibited increased expression in the CIMP-H subgroup **(Figure S4)**.

**Differential analysis of DNA methylation and gene expression**

The DNA methylation profiles of 38 paired samples sourced from TCGA were subjected to differential methylation analysis utilizing the criteria of |difference in methylation level| > 0.2 and FDR < 0.05. This analysis yielded 18,504 hypermethylated and 14,789 hypomethylated CpG sites associated with colon cancer, corresponding to 3,106 hypermethylated and 3,973 hypomethylated genes, respectively **(Figure S5)**. Furthermore, we analyzed the distribution of differentially methylated CpG sites within various genomic regions. From a functional genomics perspective, CpG sites located in proximal promoters (TSS200, TSS1500, 5'-UTRs, and first exons regions) exhibited dominance of hypermethylation, while those farther from the promoter (gene bodies, 3'-UTRs, and intergenic regions) displayed a predilection for hypomethylation **(Figure S6A)**. In addition, the majority of hypermethylated sites were situated in CpG island regions, while hypomethylated sites were more prevalent in open-sea regions **(Figure S6B)**. When considering both aspects, 4,022 CpG sites were hypermethylated, whereas only 22 CpG sites were hypomethylated in the CpG islands of the promoter regions **(Table S4)**. This pattern of genome-wide hypomethylation and specific hypermethylation in promoter CpG islands is a recurring characteristic observed in various solid tumors. Subsequently, we conducted a differential expression analysis based on the expression profiles of 26 paired patients from the TCGA dataset. This analysis, utilizing the criteria |log2 (fold change)| > 1 and FDR < 0.01, led to the identification of 1,521 highly expressed ("upregulated") and 2,171 genes with low expression ("downregulated") associated with colon cancer **(Figure S5B)**.

**Effect of methylation on cis- and trans-regulation of gene expression**

We then investigated the intersection between genes exhibiting differential expression and those showing differential methylation, specifically focusing on the promoter methylation level. Genes were categorized as "hypomethylated" if they displayed a relatively lower methylation level in colon cancer patients than in healthy controls, as measured by at least one CpG site in the promoter region. Conversely, genes were classified as "hypermethylated" if they possessed at least one hypermethylated CpG site in the promoter region. Through analysis of promoter methylation alterations, a total of 1,577 hypomethylated genes and 1,563 hypermethylated genes were identified. Among them, 793 genes demonstrated concurrent differential methylation and differential expression, encompassing 384 genes that were hypermethylated and had low expression, 103 genes that were hypomethylated and had high expression, 98 genes that were hypermethylated and had high expression, and 172 genes that were hypomethylated and had low expression **(Figure S6C)**.

To investigate the impact of DNA methylation on the expression of the same gene (cis-regulation), we conducted Pearson correlation analysis of the entire genome. Regarding the functional distribution of the genome, the cis-regulation of gene expression via DNA methylation was predominantly negatively correlated with proximal promoters (TSS200, TSS1500, 5'-UTRs, and first exons regions) and was positively correlated with regions distal to the promoter (gene body, 3'-UTRs, and intergenic regions), as detailed in **Table S5**. Notably, cis-regulation was chiefly characterized by a negative correlation between DNA methylation and gene expression within CpG island and shore regions. In contrast, in the shelves and open sea regions, the negative and positive cis-correlations were approximately equal. As the probes extended further from the CpG islands, the proportion of negative cis-correlations decreased, while the proportion of positive cis-correlations increased **(Table S6)**. In addition, through Pearson correlation analysis, we explored the relationship between the methylation of one gene and the expression of another gene, a phenomenon known as trans-regulation. Our analysis focused on a dataset comprising 3,692 genes that exhibited differential expression and 2,253 promoter CpG sites originating from a subset of 793 genes. These genes were found to be both differentially methylated and differentially expressed concurrently. Our examination of the correlation between the methylation levels of these differentially methylated genes and the expression levels of the differentially expressed genes revealed positive transregulation between hypomethylated genes and differentially expressed genes. Conversely, negative trans-regulation was observed between hypermethylated genes and differentially expressed genes **(Figure S6D)**.

**Supporting Discussion**

In the present study, we conducted a systematic investigation of genome-wide DNA methylation, somatic mutation, and gene expression profiles in colon cancer. Additionally, through a comparative analysis of colon cancer patients, normal controls, and noncolon cancer patients, we identified three CpG sites, all of which are associated with the LIFR gene and serve as colon cancer-specific diagnostic methylation biomarkers. LIFR is frequently overexpressed in numerous solid cancers, and recent studies have positioned the LIFR as a promising clinical target for cancer therapy(27). This receptor activates oncogenic signaling pathways, with JAK/STAT3 serving as immediate effectors, followed by MAPK, AKT, and mTOR downstream(28, 29). Crucially, LIFR signaling plays a pivotal role in tumor growth, progression, metastasis, stemness, and therapy resistance(30). Additionally, it modulates multiple immune cell types present in the tumor microenvironment(31). While the role of LIFR has been interpreted in tumors, its significance has mainly been recognized in precancerous lesions as a biomarker linked to iron poisoning in gastric precancerous lesions. Specifically, the LIFR gene has been identified as a biomarker associated with ferroptosis in gastric precancerous lesions(32). However, its function in colon cancer remains unexplored, and its role in tumor precancerous lesions deserves further investigation.

The methylation biomarkers demonstrated a sensitivity of approximately 89.7% and a specificity of approximately 98.2% in the prediction of colon cancer. Notably, our diagnostic methylation biomarkers exhibited a greater degree of specificity and sensitivity than most of the protein or methylation biomarkers reported in previous literature. These findings have the potential to offer fresh insights into the roles of DNA methylation in gene expression regulation, diagnosis, and the realm of colon cancer immunotherapy.

The CpG island methylator phenotype (CIMP) represents a distinct subgroup of colon cancer characterized by substantial hypermethylation of CpG sites in tumor suppressor genes. This hypermethylation leads to their inactivation and, consequently, promotes carcinogenesis (33, 34). In our study, 18.9% of patients were identified as CIMP-H. The prevalence of CIMP-H is greater in colon cancer than in other malignancies, such as papillary renal cell carcinoma (5.6%) (12), glioblastoma (8.8%) (35) and hepatocellular carcinoma (4.3%) (36). In addition, we observed a strong association between the CIMP-H subgroup and high-frequency BRAF mutations. Notably, previous research suggested that BRAF mutations might constitute early alterations in CIMP-related colon cancer by impeding the normal apoptosis of colonic epithelial cells (37). A model-based experiment demonstrated that CIMP-dependent hypermethylation and subsequent transcriptional inactivation of IGFBP7 play a role in regulating BRAF V600E-induced cellular senescence and apoptosis (38). Furthermore, mutations in SETD1B, NCOR2, and KMT2B were found to be enriched in the CIMP-H subgroup, a discovery hitherto unreported in the context of colon cancer. It is worth exploring the potential biological mechanisms underpinning these mutations in CIMP-related colon cancer. Furthermore, our findings revealed significant differences in the infiltration of immune cells and tumor mutational load among patients in different methylation subgroups, with the highest levels observed in the CIMP-H subgroup. Based on these results, we hypothesize that patients in the CIMP-H subgroup may exhibit better responses to immunotherapy.

Given the pivotal role of DNA methylation as an epigenetic mediator of gene expression, our analysis revealed a predominant pattern of negative cis-regulation in gene expression mediated by DNA methylation within proximal promoter regions. This observation aligns with the established notion that promoter hypermethylation contributes to the suppression of gene expression (39). Specifically, we found that hypermethylation of a gene within the promoter region was generally associated with a negative impact on the expression levels of other genes. In contrast, in the context of hypomethylated genes, we observed an opposite phenomenon of trans-regulation, consistent with prior findings in HCC (36). However, the underlying mechanisms responsible for these discordant correlations between hypomethylated and hypermethylated genes in trans-regulation necessitate further investigation.

**References**

1. Wei L, Jin Z, Yang S, Xu Y, Zhu Y, Ji Y. TCGA-assembler 2: software pipeline for retrieval and processing of TCGA/CPTAC data. Bioinformatics. 2018;34(9):1615-7.

2. Bibikova M, Barnes B, Tsan C, Ho V, Klotzle B, Le JM, et al. High density DNA methylation array with single CpG site resolution. Genomics. 2011;98(4):288-95.

3. Colaprico A, Silva TC, Olsen C, Garofano L, Cava C, Garolini D, et al. TCGAbiolinks: an R/Bioconductor package for integrative analysis of TCGA data. Nucleic Acids Res. 2016;44(8):e71.

4. de Ruijter TC, de Hoon JP, Slaats J, de Vries B, Janssen MJ, van Wezel T, et al. Formalin-fixed, paraffin-embedded (FFPE) tissue epigenomics using Infinium HumanMethylation450 BeadChip assays. Laboratory investigation; a journal of technical methods and pathology. 2015;95(7):833-42.

5. Kananen L, Marttila S, Nevalainen T, Jylhava J, Mononen N, Kahonen M, et al. Aging-associated DNA methylation changes in middle-aged individuals: the Young Finns study. BMC genomics. 2016;17:103.

6. Luo Y, Wong CJ, Kaz AM, Dzieciatkowski S, Carter KT, Morris SM, et al. Differences in DNA methylation signatures reveal multiple pathways of progression from adenoma to colorectal cancer. Gastroenterology. 2014;147(2):418-29 e8.

7. Timp W, Bravo HC, McDonald OG, Goggins M, Umbricht C, Zeiger M, et al. Large hypomethylated blocks as a universal defining epigenetic alteration in human solid tumors. Genome medicine. 2014;6(8):61.

8. Naumov VA, Generozov EV, Zaharjevskaya NB, Matushkina DS, Larin AK, Chernyshov SV, et al. Genome-scale analysis of DNA methylation in colorectal cancer using Infinium HumanMethylation450 BeadChips. Epigenetics. 2013;8(9):921-34.

9. McInnes T, Zou D, Rao DS, Munro FM, Phillips VL, McCall JL, et al. Genome-wide methylation analysis identifies a core set of hypermethylated genes in CIMP-H colorectal cancer. BMC cancer. 2017;17(1):228.

10. Qu X, Sandmann T, Frierson H, Jr., Fu L, Fuentes E, Walter K, et al. Integrated genomic analysis of colorectal cancer progression reveals activation of EGFR through demethylation of the EREG promoter. Oncogene. 2016;35(50):6403-15.

11. Zhao M, Kim P, Mitra R, Zhao J, Zhao Z. TSGene 2.0: an updated literature-based knowledgebase for tumor suppressor genes. Nucleic Acids Res. 2016;44(D1):D1023-31.

12. Cancer Genome Atlas Research N, Linehan WM, Spellman PT, Ricketts CJ, Creighton CJ, Fei SS, et al. Comprehensive Molecular Characterization of Papillary Renal-Cell Carcinoma. N Engl J Med. 2016;374(2):135-45.

13. Li G, Xu W, Zhang L, Liu T, Jin G, Song J, et al. Development and validation of a CIMP-associated prognostic model for hepatocellular carcinoma. EBioMedicine. 2019;47:128-41.

14. Wilkerson MD, Hayes DN. ConsensusClusterPlus: a class discovery tool with confidence assessments and item tracking. Bioinformatics. 2010;26(12):1572-3.

15. Breiman L. Random forests. Machine Learning. 2001;45(1):5-32.

16. Mayakonda A, Lin DC, Assenov Y, Plass C, Koeffler HP. Maftools: efficient and comprehensive analysis of somatic variants in cancer. Genome Res. 2018;28(11):1747-56.

17. Chalmers ZR, Connelly CF, Fabrizio D, Gay L, Ali SM, Ennis R, et al. Analysis of 100,000 human cancer genomes reveals the landscape of tumor mutational burden. Genome medicine. 2017;9(1):34.

18. Li T, Fu J, Zeng Z, Cohen D, Li J, Chen Q, et al. TIMER2.0 for analysis of tumor-infiltrating immune cells. Nucleic acids research. 2020;48(W1):W509-w14.

19. Aran D, Hu Z, Butte AJ. xCell: digitally portraying the tissue cellular heterogeneity landscape. Genome biology. 2017;18(1):220.

20. Racle J, de Jonge K, Baumgaertner P, Speiser DE, Gfeller D. Simultaneous enumeration of cancer and immune cell types from bulk tumor gene expression data. Elife. 2017;6.

21. Newman AM, Liu CL, Green MR, Gentles AJ, Feng W, Xu Y, et al. Robust enumeration of cell subsets from tissue expression profiles. Nature methods. 2015;12(5):453-7.

22. Fortin JP, Triche TJ, Jr., Hansen KD. Preprocessing, normalization and integration of the Illumina HumanMethylationEPIC array with minfi. Bioinformatics. 2017;33(4):558-60.

23. Sandoval J, Mendez-Gonzalez J, Nadal E, Chen G, Carmona FJ, Sayols S, et al. A prognostic DNA methylation signature for stage I non-small-cell lung cancer. J Clin Oncol. 2013;31(32):4140-7.

24. Price ME, Cotton AM, Lam LL, Farre P, Emberly E, Brown CJ, et al. Additional annotation enhances potential for biologically-relevant analysis of the Illumina Infinium HumanMethylation450 BeadChip array. Epigenetics Chromatin. 2013;6(1):4.

25. Chen YA, Lemire M, Choufani S, Butcher DT, Grafodatskaya D, Zanke BW, et al. Discovery of cross-reactive probes and polymorphic CpGs in the Illumina Infinium HumanMethylation450 microarray. Epigenetics. 2013;8(2):203-9.

26. Love MI, Huber W, Anders S. Moderated estimation of fold change and dispersion for RNA-seq data with DESeq2. Genome biology. 2014;15(12):550.

27. Viswanadhapalli S, Dileep KV, Zhang KYJ, Nair HB, Vadlamudi RK. Targeting LIF/LIFR signaling in cancer. Genes Dis. 2022;9(4):973-80.

28. Hara T, Chanoch-Myers R, Mathewson ND, Myskiw C, Atta L, Bussema L, et al. Interactions between cancer cells and immune cells drive transitions to mesenchymal-like states in glioblastoma. Cancer Cell. 2021;39(6):779-92 e11.

29. Shao J, Zhu W, Ding Y, Zhu H, Jing X, Yu H, et al. Phosphorylation of LIFR promotes prostate cancer progression by activating the AKT pathway. Cancer Lett. 2019;451:110-21.

30. Lin SR, Wen YC, Yeh HL, Jiang KC, Chen WH, Mokgautsi N, et al. EGFR-upregulated LIFR promotes SUCLG2-dependent castration resistance and neuroendocrine differentiation of prostate cancer. Oncogene. 2020;39(44):6757-75.

31. Zhang F, Wang Y, Li H, Li L, Yang X, You X, et al. Pan-cancer analysis identifies LIFR as a prognostic and immunological biomarker for uterine corpus endometrial carcinoma. Front Oncol. 2023;13:1118906.

32. Kuang Y, Yang K, Meng L, Mao Y, Xu F, Liu H. Identification and validation of ferroptosis-related biomarkers and the related pathogenesis in precancerous lesions of gastric cancer. Sci Rep. 2023;13(1):16074.

33. Advani SM, Advani P, DeSantis SM, Brown D, VonVille HM, Lam M, et al. Clinical, Pathological, and Molecular Characteristics of CpG Island Methylator Phenotype in Colorectal Cancer: A Systematic Review and Meta-analysis. Translational oncology. 2018;11(5):1188-201.

34. Toyota M, Ahuja N, Ohe-Toyota M, Herman JG, Baylin SB, Issa JP. CpG island methylator phenotype in colorectal cancer. Proc Natl Acad Sci U S A. 1999;96(15):8681-6.

35. Noushmehr H, Weisenberger DJ, Diefes K, Phillips HS, Pujara K, Berman BP, et al. Identification of a CpG island methylator phenotype that defines a distinct subgroup of glioma. Cancer cell. 2010;17(5):510-22.

36. Cheng J, Wei D, Ji Y, Chen L, Yang L, Li G, et al. Integrative analysis of DNA methylation and gene expression reveals hepatocellular carcinoma-specific diagnostic biomarkers. Genome medicine. 2018;10(1):42.

37. Hughes LA, Khalid-de Bakker CA, Smits KM, van den Brandt PA, Jonkers D, Ahuja N, et al. The CpG island methylator phenotype in colorectal cancer: progress and problems. Biochim Biophys Acta. 2012;1825(1):77-85.

38. Hinoue T, Weisenberger DJ, Pan F, Campan M, Kim M, Young J, et al. Analysis of the association between CIMP and BRAF in colorectal cancer by DNA methylation profiling. Plos One. 2009;4(12):e8357.

39. Jones PA. Functions of DNA methylation: islands, start sites, gene bodies and beyond. Nat Rev Genet. 2012;13(7):484-92.

**Supporting Figures**

**
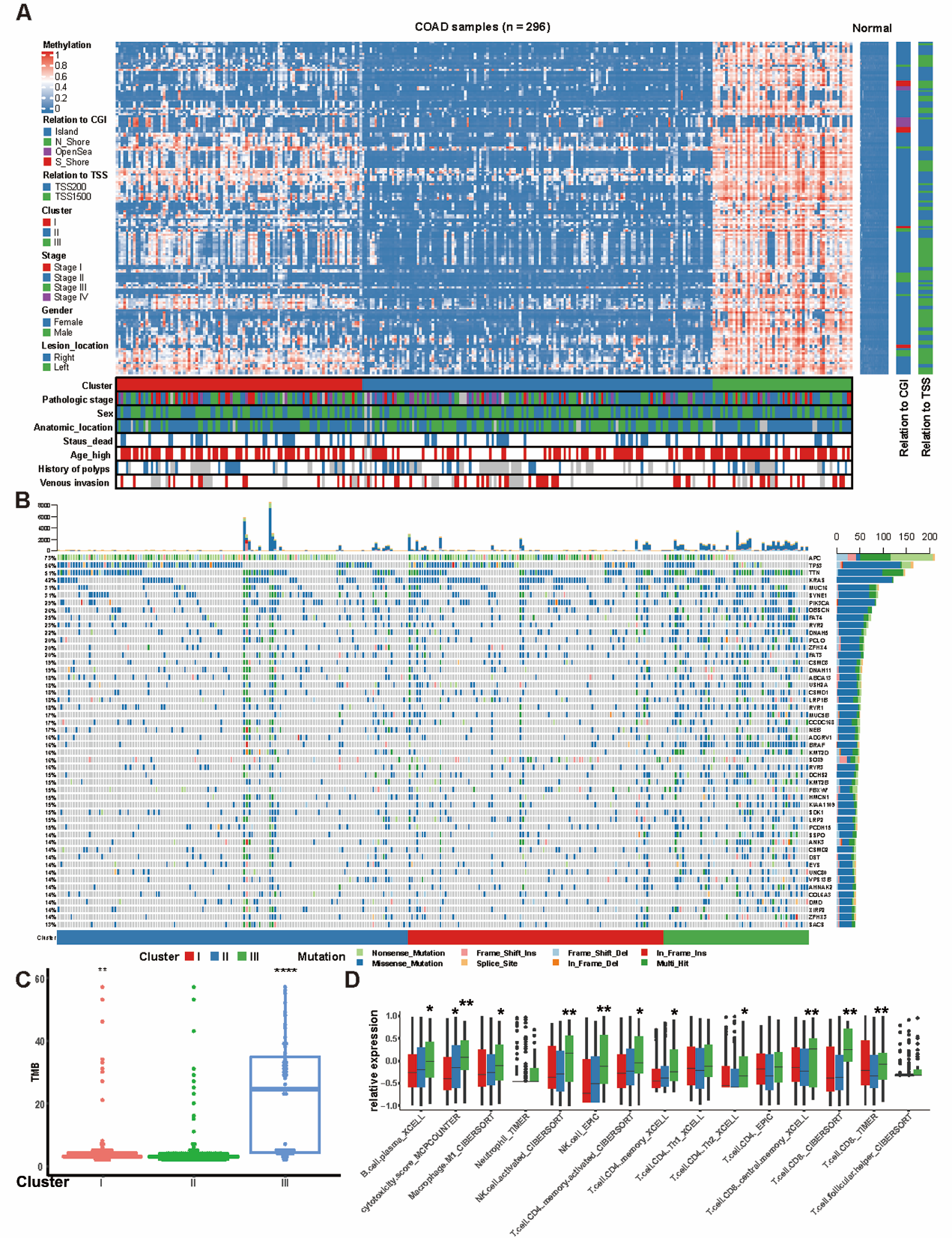
**

**Figure S1.** The DNA methylation landscape of colon cancer. **(A)** Three methylation clusters were identified based on k-means consensus clustering. The rows are 171 CpG sites that exhibited high variation (SD > 0.2) in tumor tissues and low (β value < 0.05) methylation in normal tissues. Cluster 3 (green) exhibited a hypermethylation pattern at almost all CpG sites and was considered the CpG island methylator phenotype-high (CIMP-H) subgroup. **(B)** Oncoplots for the top 60 mutated genes in the three methylation clusters are shown in order of descending frequency. Each column represents a patient. The rows represent genes. The colored bars represent mutations as described in the legend. The gray bars represent the genes with no mutations in a given tumor. **(C)** Tumor mutation burden according to methylation cluster in colon cancer. The Mann–Whitney test was applied to evaluate the significance of differences in means between two subgroups, and the Kruskal‒Wallis test was used for all three subgroups. ***P* < 0.01; **** *P* < 0.0001. **(D)** Comparison of the infiltration of multiple immune cells in the three methylation clusters, as estimated by multiple methods based on RNA sequencing data; *, P<0.05; **, P<0.01, compared with Cluster 1. Mann–Whitney test.


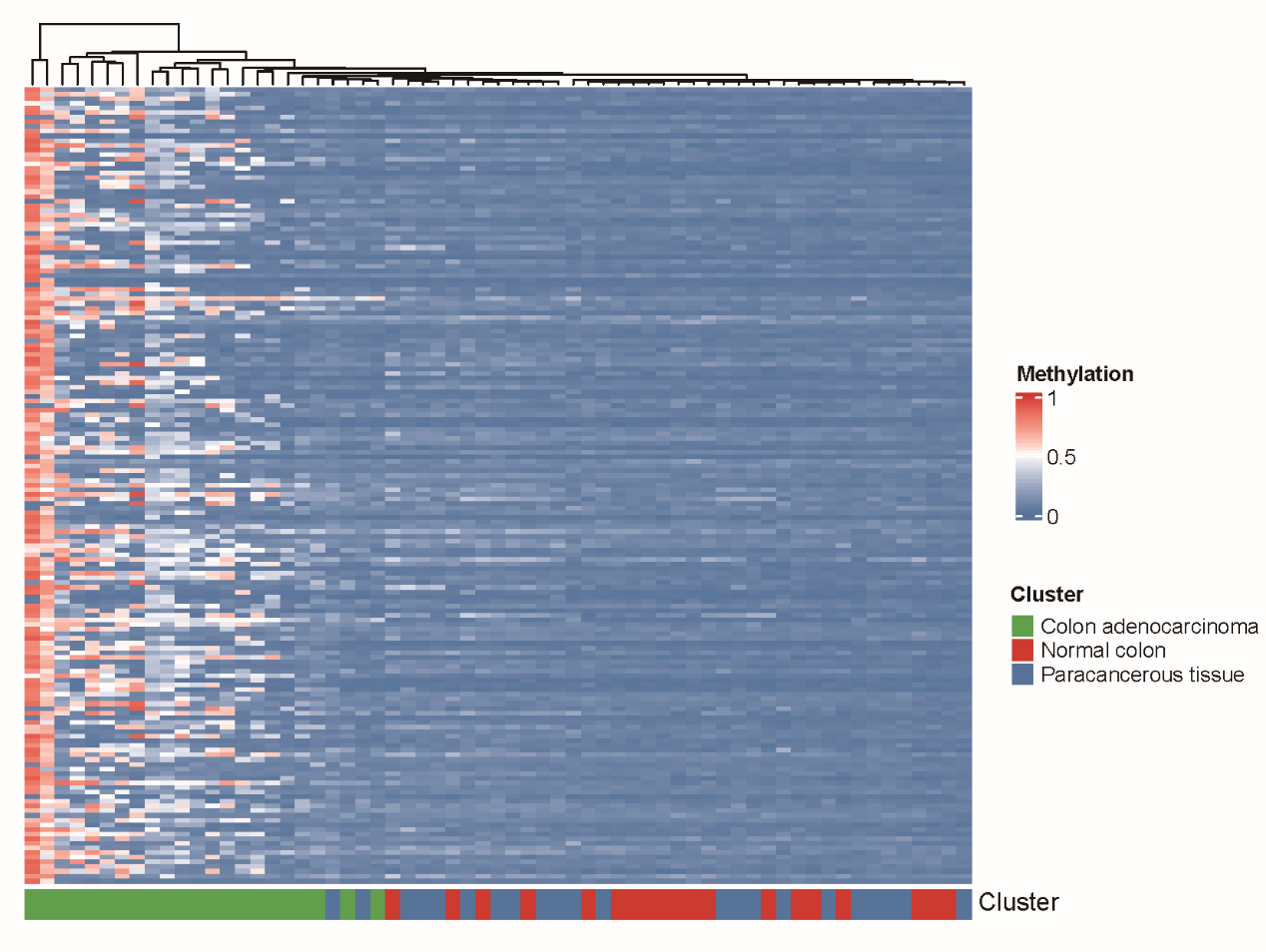


**Figure S2.** Three methylation clusters were identified based on the k-means consensus clustering in GSE42752 colon cancer dataset.


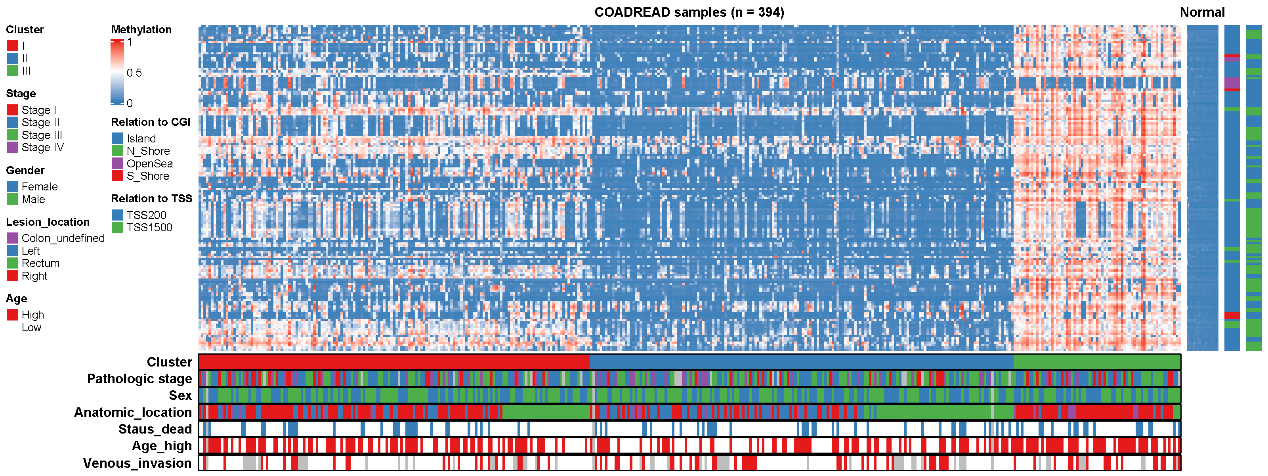


**Figure S3.** The DNA methylation landscape of colorectal cancer. Three methylation clusters were identified based on the k-means consensus clustering. Rows are 171 CpG sites that possessed high variation (SD > 0.2) in tumor tissues and low (β value < 0.05) methylation level in normal tissues. Cluster 3 (green) showed a hypermethylation pattern in almost CpG sites and was considered as the CpG island methylator phenotype-high (CIMP-H) subgroup.


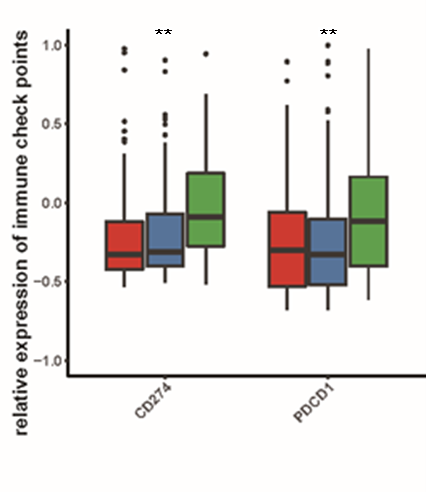


**Figure S4.** Comparison of the expression of check points in the three methylation clusters.

**
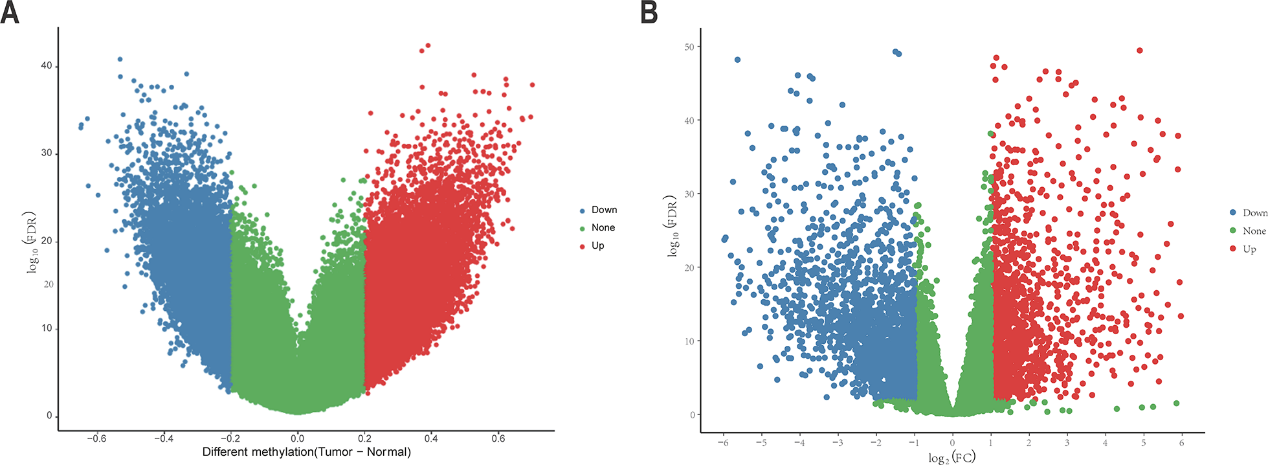
**

**Figure S5.** Volcano plot of differential methylation CpG sites and expression genes.

**(A)** A volcano plot was generated based on absolute differences of beta-value in combination with adjusted *P*-values. A total of 18, 504 hypermethylated and 14, 789 hypomethylated CpG sites were identified. **(B)** A volcano plot was generated based on absolute fold change (FC) in combination with adjusted *P*-values. A total of 1521 highly expressed and 2171 lowly expressed genes were identified.


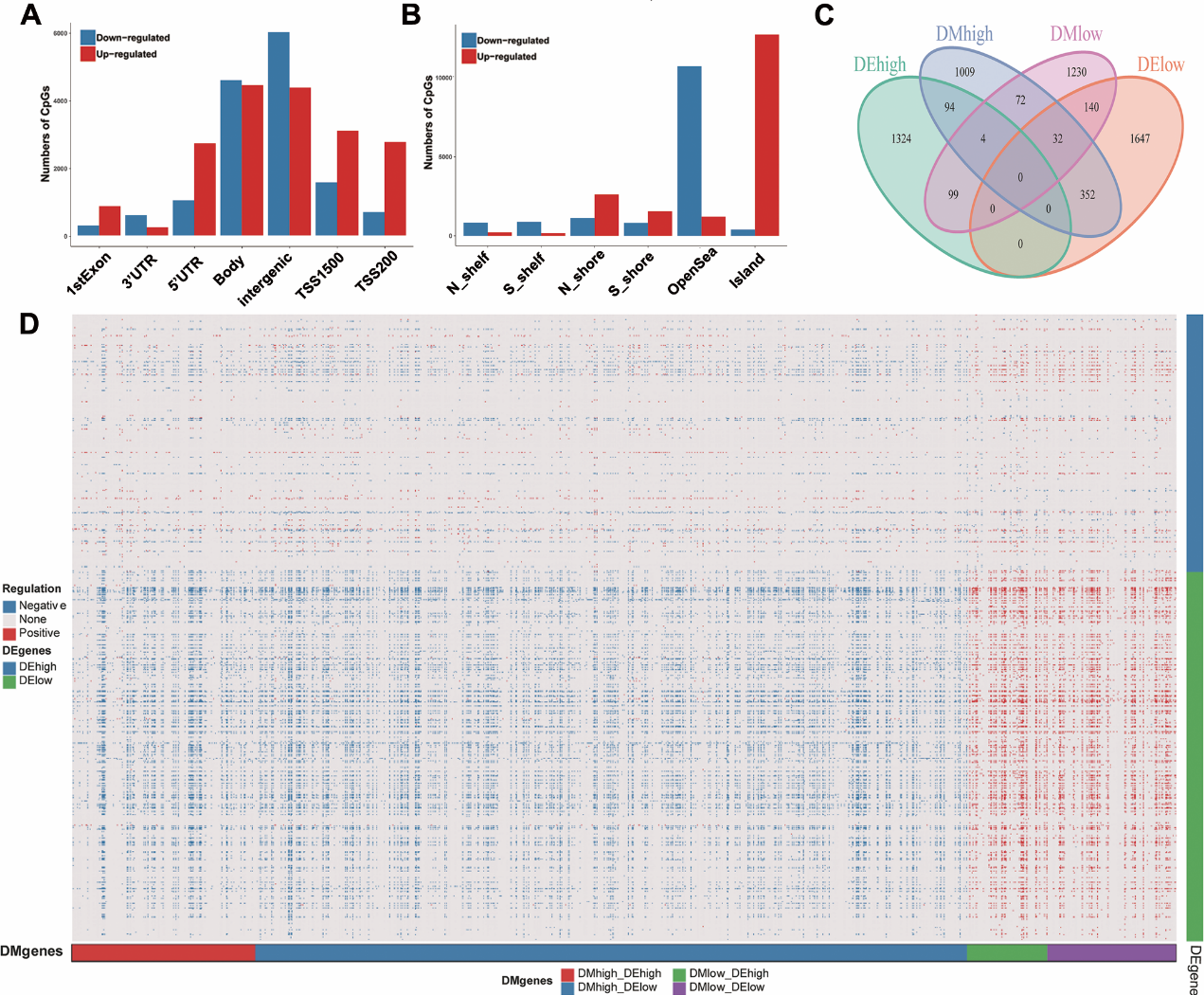


**Figure S6.** Relationship between DNA methylation and gene expression in colon cancer. **(A)** Distribution of differentially methylated CpG sites according to the distance to the TSS. **(B)** Distribution of differentially methylated CpG sites according to CpG islands. **(C)** Comparison of differentially methylated genes and differentially expressed genes. A gene was considered differentially methylated if at least one promoter CpG site was significantly differentially methylated. **(D)** Correlation between promoter methylation and another gene expression. This analysis focused on 3692 differentially expressed genes (rows), and 2253 promoter CpG sites (columns) originated from 793 genes, which were differential methylated and differential expressed, simultaneously. Positive and negative correlations are shown in red and blue, respectively.


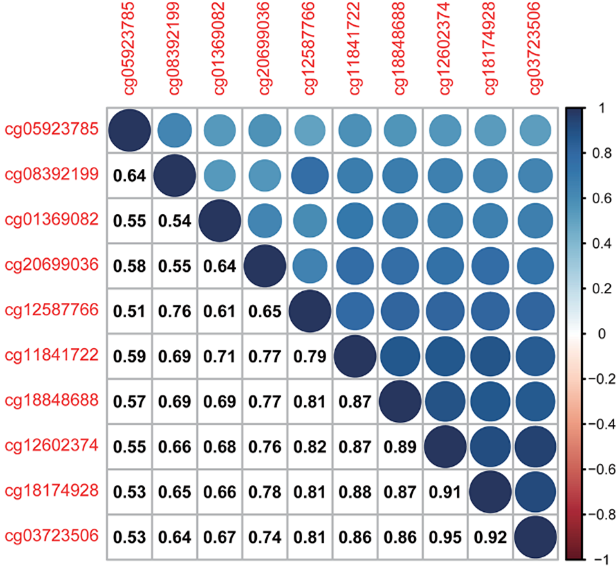


**Figure S7** co-methylated between other CpG sites in neighboring regions of three specific biomarkers. The coefficients were calculated by Pearson correlation analysis.


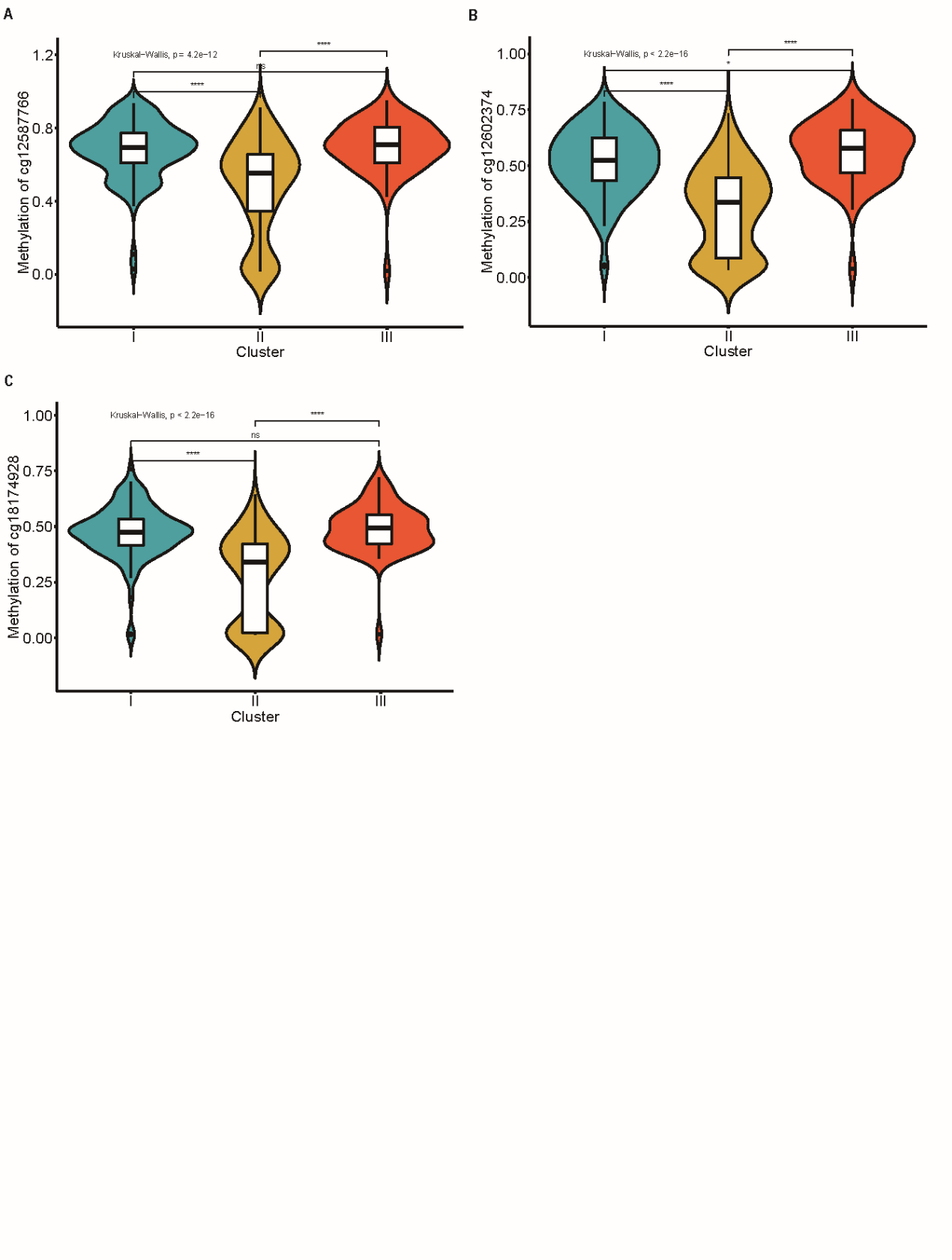


**Figure S8** CIMP related methylation of three colon cancer-specific CpG sites. The Mann–Whitney test was applied to evaluate the significant difference of means between each two subgroups and the Kruskal-Wallis test for all three subgroups. (ns: not significant; **P* < 0.05; **** *P* < 0.0001)


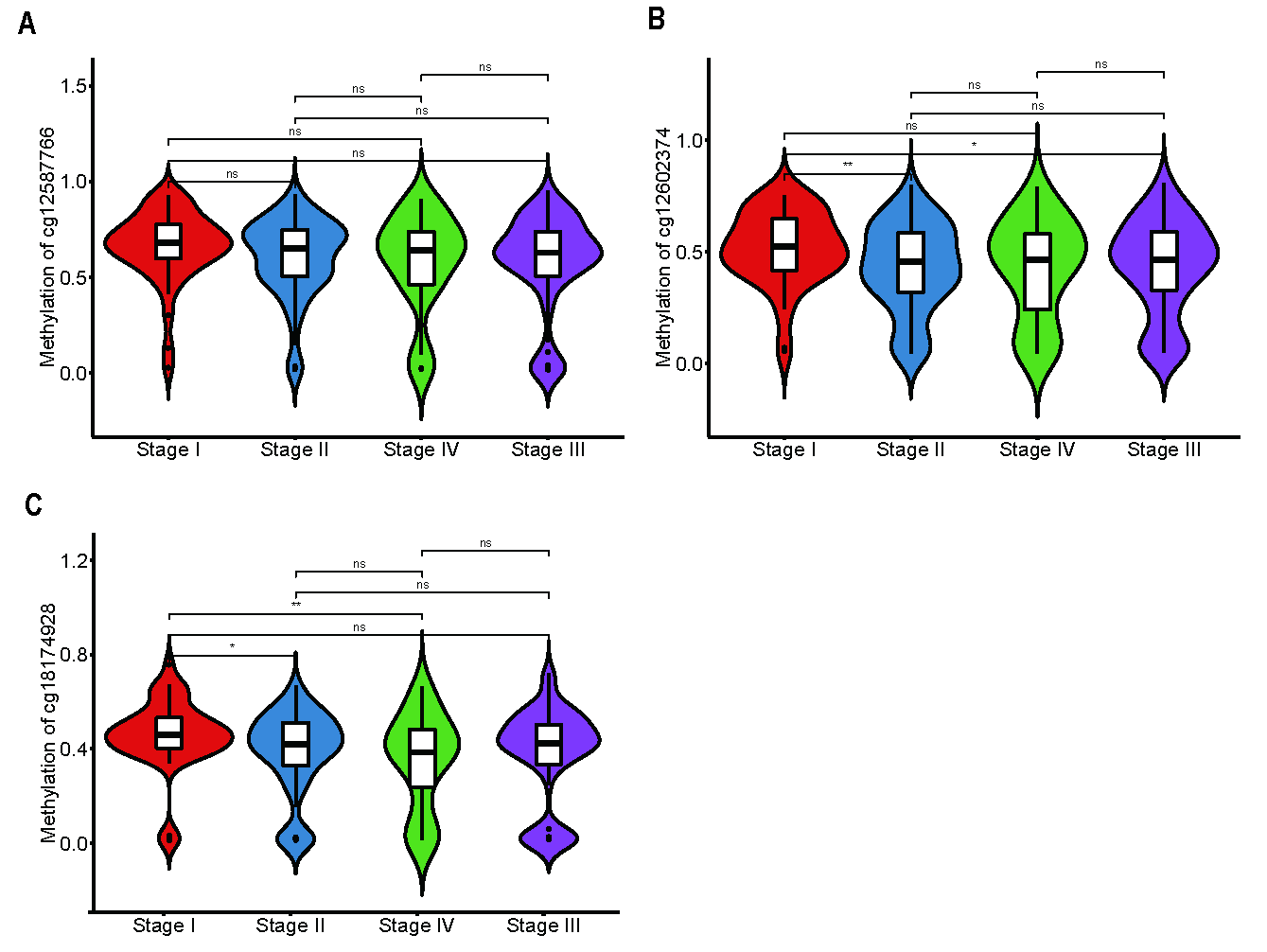


**Figure S9.** Stage related methylation of three colon cancer-specific CpG sites. The Mann–Whitney test was applied to evaluate the significant difference of means between each two subgroups and the Kruskal-Wallis test for all three subgroups. (ns: not significant; **P* < 0.05; ** *P* < 0.01)

**
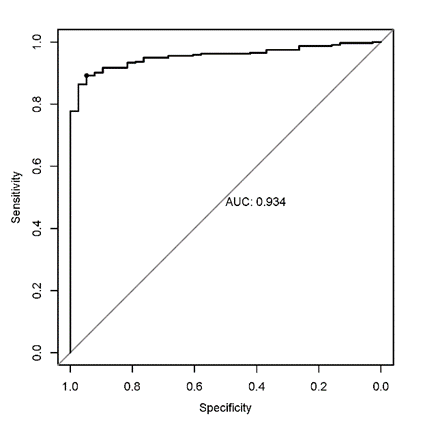
**

**Figure S10.** Performance of LIFR-related hypermethylated sites as diagnostic biomarkers of rectal cancer (TCGA-READ cohort) in TCGA datasets.

**Supporting Tables**

**Table S1** **Methylation information for 171 most variable CpG sites mapped to TSGs in the TCGA dataset.**

| **IlmnID** | **Gene_Symbol** | **Chromosome** | **Genomic_Coordinate** | **Relation_to_UCSC_CpG_Island** | **UCSC_RefGene_Group** |
| --- | --- | --- | --- | --- | --- |
| cg00922376 | PRDM2 | chr1 | 14026584 | Island | TSS200 |
| cg07162257 | ALPL | chr1 | 21835692 | Island | TSS200 |
| cg09763175 | PRKAA2 | chr1 | 57110722 | Island | TSS1500 |
| cg11018723 | RUNX3 | chr1 | 25256939 | Island | TSS200 |
| cg12690148 | FOXD3 | chr1 | 63788429 | Island | TSS1500 |
| cg13554489 | ALPL | chr1 | 21835685 | Island | TSS200 |
| cg19083779 | PRKAA2 | chr1 | 57110724 | Island | TSS1500 |
| cg22154616 | ALPL | chr1 | 21835703 | Island | TSS200 |
| cg22902824 | CHD5 | chr1 | 6240271 | Island | TSS200 |
| cg24151926 | CHD5 | chr1 | 6240455 | Island | TSS1500 |
| cg26421310 | RUNX3 | chr1 | 25257058 | Island | TSS1500 |
| cg00522056 | RPRM | chr2 | 154335386 | Island | TSS200 |
| cg08119884 | CTNNA2 | chr2 | 79739863 | Island | TSS200 |
| cg10639888 | RPRM | chr2 | 154335368 | Island | TSS200 |
| cg14634210 | SPTBN1 | chr2 | 54682758 | N_Shore | TSS1500 |
| cg15400238 | RPRM | chr2 | 154335640 | S_Shore | TSS1500 |
| cg16882226 | CHST10 | chr2 | 101034257 | Island | TSS200 |
| cg19861138 | RPRM | chr2 | 154335384 | Island | TSS200 |
| cg20637307 | ERBB4 | chr2 | 213403960 | S_Shore | TSS1500 |
| cg21338532 | RPRM | chr2 | 154335348 | Island | TSS200 |
| cg07101782 | MLH1 | chr3 | 37034495 | Island | TSS1500 |
| cg12479047 | RARB | chr3 | 25469573 | OpenSea | TSS200 |
| cg20899354 | RARB | chr3 | 25469392 | OpenSea | TSS1500 |
| cg06269673 | SHISA3 | chr4 | 42399792 | Island | TSS200 |
| cg12828819 | FAT4 | chr4 | 126236816 | Island | TSS1500 |
| cg13740698 | SHISA3 | chr4 | 42399384 | Island | TSS1500 |
| cg14866200 | SHISA3 | chr4 | 42399843 | Island | TSS200 |
| cg16862295 | SHISA3 | chr4 | 42399798 | Island | TSS200 |
| cg00281842 | PCDHGC3 | chr5 | 140855562 | Island | TSS200 |
| cg02033258 | PDLIM4 | chr5 | 131593261 | Island | TSS200 |
| cg02430347 | PCDHGC3 | chr5 | 140855547 | Island | TSS200 |
| cg03667968 | APC | chr5 | 112073438 | OpenSea | TSS200 |
| cg07234569 | BASP1 | chr5 | 17216679 | Island | TSS1500 |
| cg08391321 | MCC | chr5 | 112630695 | S_Shore | TSS200 |
| cg11613015 | APC | chr5 | 112073406 | OpenSea | TSS200 |
| cg14511739 | APC | chr5 | 112073373 | OpenSea | TSS200 |
| cg16485558 | RNF180 | chr5 | 63461566 | Island | TSS200 |
| cg22035501 | APC | chr5 | 112073398 | OpenSea | TSS200 |
| cg22638593 | PDLIM4 | chr5 | 131593259 | Island | TSS200 |
| cg23359276 | PCDHGC3 | chr5 | 140855504 | Island | TSS200 |
| cg24994173 | TSLP | chr5 | 110408997 | Island | TSS200 |
| cg25203704 | BASP1 | chr5 | 17216922 | Island | TSS1500 |
| cg01327552 | RPS6KA2 | chr6 | 167275809 | Island | TSS200 |
| cg04508687 | RPS6KA2 | chr6 | 167275843 | Island | TSS200 |
| cg07015190 | RPS6KA2 | chr6 | 167276275 | Island | TSS1500 |
| cg09184730 | RPS6KA2 | chr6 | 167275834 | Island | TSS200 |
| cg09987129 | RPS6KA2 | chr6 | 167276419 | Island | TSS1500 |
| cg14017655 | EYA4 | chr6 | 133562193 | Island | TSS1500 |
| cg14343214 | EYA4 | chr6 | 133562056 | N_Shore | TSS1500 |
| cg20206437 | RPS6KA2 | chr6 | 167276359 | Island | TSS1500 |
| cg21760146 | RPS6KA2 | chr6 | 167275781 | Island | TSS200 |
| cg24699519 | RPS6KA2 | chr6 | 167275832 | Island | TSS200 |
| cg02215070 | AKR1B1 | chr7 | 134144055 | Island | TSS200 |
| cg07589773 | IKZF1 | chr7 | 50343883 | Island | TSS1500 |
| cg07871590 | LRRC4 | chr7 | 127671193 | Island | TSS200 |
| cg16132520 | AKR1B1 | chr7 | 134143906 | Island | TSS200 |
| cg18607529 | IKZF1 | chr7 | 50343869 | Island | TSS1500 |
| cg20078466 | IKZF1 | chr7 | 50344331 | Island | TSS200 |
| cg21079345 | AKR1B1 | chr7 | 134144036 | Island | TSS200 |
| cg07233097 | SOX7 | chr8 | 10588040 | Island | TSS200 |
| cg22008625 | SOX7 | chr8 | 10588386 | Island | TSS1500 |
| cg00329154 | EBF3 | chr10 | 131762598 | Island | TSS1500 |
| cg05937453 | SFRP5 | chr10 | 99531765 | Island | TSS200 |
| cg06048524 | CXCL12 | chr10 | 44880542 | Island | TSS200 |
| cg07834955 | SFRP5 | chr10 | 99531879 | Island | TSS200 |
| cg10927719 | CXCL12 | chr10 | 44880819 | Island | TSS1500 |
| cg17267805 | CXCL12 | chr10 | 44880545 | Island | TSS200 |
| cg17820890 | SFRP5 | chr10 | 99531790 | Island | TSS200 |
| cg22831607 | SFRP5 | chr10 | 99531797 | Island | TSS200 |
| cg26267854 | CXCL12 | chr10 | 44880562 | Island | TSS200 |
| cg01547141 | SCUBE2 | chr11 | 9113152 | Island | TSS200 |
| cg02783889 | SCUBE2 | chr11 | 9113370 | Island | TSS1500 |
| cg05091519 | PAX6 | chr11 | 31839552 | Island | TSS200 |
| cg09719477 | ZBTB16 | chr11 | 113930430 | Island | TSS1500 |
| cg10376408 | SCUBE2 | chr11 | 9113406 | Island | TSS1500 |
| cg10782380 | SCUBE2 | chr11 | 9113167 | Island | TSS200 |
| cg12086936 | PAX6 | chr11 | 31832879 | Island | TSS200 |
| cg14002345 | PAX6 | chr11 | 31833016 | Island | TSS200 |
| cg14906390 | ZBTB16 | chr11 | 113930251 | Island | TSS200 |
| cg15778437 | PAX6 | chr11 | 31839521 | Island | TSS200 |
| cg00470794 | CHFR | chr12 | 133464685 | Island | TSS1500 |
| cg02667335 | CHFR | chr12 | 133464515 | Island | TSS1500 |
| cg04774496 | CHFR | chr12 | 133464638 | Island | TSS1500 |
| cg07951978 | CHFR | chr12 | 133464728 | Island | TSS1500 |
| cg12551582 | CHFR | chr12 | 133464601 | Island | TSS1500 |
| cg16087447 | PRICKLE1 | chr12 | 42983805 | Island | TSS1500 |
| cg18533833 | CHFR | chr12 | 133464613 | Island | TSS1500 |
| cg19313015 | CHFR | chr12 | 133464323 | Island | TSS200 |
| cg21273703 | CHFR | chr12 | 133464351 | Island | TSS200 |
| cg21645164 | CHFR | chr12 | 133464327 | Island | TSS200 |
| cg24400921 | PRICKLE1 | chr12 | 42984337 | Island | TSS1500 |
| cg25734490 | ASCL1 | chr12 | 103351188 | N_Shore | TSS1500 |
| cg26394825 | CHFR | chr12 | 133464689 | Island | TSS1500 |
| cg26410484 | CHFR | chr12 | 133464646 | Island | TSS1500 |
| cg26490054 | SLC5A8 | chr12 | 101604056 | S_Shore | TSS200 |
| cg26832509 | CHFR | chr12 | 133464855 | Island | TSS1500 |
| cg26866482 | ASCL1 | chr12 | 103351443 | N_Shore | TSS200 |
| cg27382164 | CHFR | chr12 | 133464737 | Island | TSS1500 |
| cg03036557 | GPC5 | chr13 | 92050720 | N_Shore | TSS1500 |
| cg05855588 | KL | chr13 | 33590273 | Island | TSS1500 |
| cg08999807 | GPC5 | chr13 | 92050776 | N_Shore | TSS200 |
| cg10548038 | GPC5 | chr13 | 92050731 | N_Shore | TSS1500 |
| cg12678562 | GPC5 | chr13 | 92050726 | N_Shore | TSS1500 |
| cg14145477 | KL | chr13 | 33590493 | Island | TSS200 |
| cg04627496 | NTRK3 | chr15 | 88799973 | Island | TSS1500 |
| cg09948076 | SYNM | chr15 | 99645088 | Island | TSS200 |
| cg10637512 | SYNM | chr15 | 99645096 | Island | TSS200 |
| cg12664560 | HOMER2 | chr15 | 83621517 | Island | TSS200 |
| cg19890277 | ALDH1A2 | chr15 | 58358384 | Island | TSS1500 |
| cg27569300 | SYNM | chr15 | 99645065 | Island | TSS1500 |
| cg00262031 | NDRG4 | chr16 | 58498574 | Island | TSS200 |
| cg00735962 | PRKCB | chr16 | 23847240 | Island | TSS200 |
| cg00984694 | NDRG4 | chr16 | 58498151 | Island | TSS1500 |
| cg04797985 | NDRG4 | chr16 | 58498190 | Island | TSS1500 |
| cg05151621 | CBFA2T3 | chr16 | 89008406 | Island | TSS1500 |
| cg06650115 | NDRG4 | chr16 | 58498585 | Island | TSS200 |
| cg08406370 | PRKCB | chr16 | 23846893 | N_Shore | TSS1500 |
| cg11306587 | NDRG4 | chr16 | 58497714 | Island | TSS1500 |
| cg13031432 | NDRG4 | chr16 | 58497767 | Island | TSS1500 |
| cg00815093 | HIC1 | chr17 | 1957390 | Island | TSS1500 |
| cg00958884 | CYGB | chr17 | 74533976 | Island | TSS200 |
| cg02396496 | CYGB | chr17 | 74534090 | Island | TSS1500 |
| cg09633973 | HIC1 | chr17 | 1957365 | Island | TSS1500 |
| cg11144056 | HIC1 | chr17 | 1957410 | Island | TSS1500 |
| cg17029019 | HIC1 | chr17 | 1959124 | Island | TSS1500 |
| cg17299935 | CYGB | chr17 | 74534077 | Island | TSS1500 |
| cg17416280 | HIC1 | chr17 | 1957394 | Island | TSS1500 |
| cg17739038 | HIC1 | chr17 | 1957496 | Island | TSS1500 |
| cg19249708 | CYGB | chr17 | 74533969 | Island | TSS200 |
| cg25432975 | HIC1 | chr17 | 1959066 | Island | TSS1500 |
| cg00854242 | GALR1 | chr18 | 74961724 | Island | TSS1500 |
| cg06562865 | GALR1 | chr18 | 74961451 | N_Shore | TSS1500 |
| cg12967001 | EPB41L3 | chr18 | 5544089 | Island | TSS200 |
| cg14352983 | L3MBTL4 | chr18 | 6414976 | S_Shore | TSS200 |
| cg15146859 | GALR1 | chr18 | 74961737 | Island | TSS1500 |
| cg16452086 | NFATC1 | chr18 | 77160030 | Island | TSS1500 |
| cg16622495 | EPB41L3 | chr18 | 5544099 | Island | TSS200 |
| cg16924702 | EPB41L3 | chr18 | 5544231 | Island | TSS1500 |
| cg18556788 | L3MBTL4 | chr18 | 6414974 | S_Shore | TSS200 |
| cg22335490 | EPB41L3 | chr18 | 5544237 | Island | TSS1500 |
| cg23482397 | TCF4 | chr18 | 53257226 | Island | TSS1500 |
| cg23564700 | EPB41L3 | chr18 | 5544169 | Island | TSS200 |
| cg26721264 | GALR1 | chr18 | 74961727 | Island | TSS1500 |
| cg18872321 | GATA5 | chr20 | 61051751 | Island | TSS1500 |
| cg22152407 | THBD | chr20 | 23030446 | Island | TSS200 |
| cg24500900 | GATA5 | chr20 | 61051423 | Island | TSS1500 |
| cg25397597 | THBD | chr20 | 23030442 | Island | TSS200 |
| cg25667841 | GATA5 | chr20 | 61051762 | Island | TSS1500 |
| cg22737001 | RUNX3 | chr1 | 25257029 | Island | TSS1500 |
| cg00143045 | RPRM | chr2 | 154335593 | S_Shore | TSS1500 |
| cg13218903 | RPRM | chr2 | 154335379 | Island | TSS200 |
| cg03497419 | MLH1 | chr3 | 37034654 | Island | TSS200 |
| cg06108510 | MLH1 | chr3 | 37035063 | Island | TSS1500 |
| cg07064226 | MLH1 | chr3 | 37034997 | Island | TSS1500 |
| cg27586588 | MLH1 | chr3 | 37034661 | Island | TSS200 |
| cg00577935 | APC | chr5 | 112073348 | OpenSea | TSS1500 |
| cg02780295 | PCDHGC3 | chr5 | 140855463 | Island | TSS200 |
| cg04614008 | MCC | chr5 | 112630677 | S_Shore | TSS200 |
| cg13993336 | PCDHGC3 | chr5 | 140855482 | Island | TSS200 |
| cg24003542 | MCC | chr5 | 112630684 | S_Shore | TSS200 |
| cg14839257 | RPS6KA2 | chr6 | 167275999 | Island | TSS1500 |
| cg15063355 | CHFR | chr12 | 133464596 | Island | TSS1500 |
| cg23653008 | CHFR | chr12 | 133464419 | Island | TSS1500 |
| cg24727133 | CHFR | chr12 | 133464594 | Island | TSS1500 |
| cg27084026 | CHFR | chr12 | 133464496 | Island | TSS1500 |
| cg13744663 | GPC5 | chr13 | 92050675 | N_Shore | TSS1500 |
| cg14012112 | PCDH9 | chr13 | 67804637 | Island | TSS200 |
| cg25422943 | PCDH9 | chr13 | 67804733 | Island | TSS1500 |
| cg23882658 | HIC1 | chr17 | 1959132 | Island | TSS1500 |
| cg18961944 | GALR1 | chr18 | 74961360 | N_Shore | TSS1500 |
| cg21661027 | GALR1 | chr18 | 74961342 | N_Shore | TSS1500 |
|  |  |  |  |  |  |

**Table S2 Characteristics of patients generated from TCGA in three methylation subgroups.**

| **Parameters** | **I** | **II** | **III** | ***p*** |
| --- | --- | --- | --- | --- |
| Numbers | 99 | 141 | 56 |  |
| Age = Low (%) | 45 (45.5) | 81 (58.3) | 15 (26.8) | <0.001 |
| Anatomic_location = Right (%) | 70 (72.9) | 60 (45.1) | 46 (92.0) | <0.001 |
| gender = Male (%) | 54 (54.5) | 79 (56.8) | 25 (44.6) | 0.297 |
| history_of_colon_polyps = Yes (%) | 21 (26.6) | 20 (19.6) | 12 (27.3) | 0.444 |
| pathologic_M (%) |  |  |  | 0.033 |
| M0 | 63 (65.6) | 92 (67.2) | 43 (76.8) |  |
| M1 | 10 (10.4) | 27 (19.7) | 4 (7.1) |  |
| MX | 23 (24.0) | 18 (13.1) | 9 (16.1) |  |
| pathologic_N (%) |  |  |  | 0.665 |
| N0 | 59 (59.6) | 76 (54.7) | 36 (64.3) |  |
| N1 | 22 (22.2) | 40 (28.8) | 12 (21.4) |  |
| N2 | 18 (18.2) | 23 (16.5) | 8 (14.3) |  |
| pathologic_T (%) |  |  |  | 0.076 |
| T1+T2 | 23 (23.5) | 15 (10.8) | 12 (21.4) |  |
| T3 | 60 (61.2) | 106 (76.3) | 37 (66.1) |  |
| T4 | 15 (15.3) | 18 (12.9) | 7 (12.5) |  |
| pathologic_stage (%) |  |  |  | 0.032 |
| Stage I | 21 (21.9) | 12 (9.0) | 11 (19.6) |  |
| Stage II | 35 (36.5) | 56 (42.1) | 24 (42.9) |  |
| Stage III | 30 (31.2) | 38 (28.6) | 17 (30.4) |  |
| Stage IV | 10 (10.4) | 27 (20.3) | 4 (7.1) |  |
| venous_invasion = Yes (%) | 15 (17.6) | 31 (25.4) | 14 (28.6) | 0.276 |
| vital_status = Dead (%) | 25 (25.3) | 30 (21.6) | 15 (26.8) | 0.681 |
|  |  |  |  |  |

P-values were calculated by Fisher's exact test.

**Table S3 Distribution of significantly mutated genes in three methylation subgroups.**

| **Gene** | **I** | **II** | **III** | ***P*** |
| --- | --- | --- | --- | --- |
| *BRAF* | 6.06 | 5.88 | 60.71 | <0.001 |
| *SETD1B* | 7.07 | 5.88 | 42.86 | <0.001 |
| *NCOR2* | 4.04 | 5.88 | 35.71 | <0.001 |
| *KMT2B* | 11.11 | 6.62 | 44.64 | <0.001 |
|  |  |  |  |  |

*P*-values were calculated by Fisher's exact test.

**Table S4: Distribution of differentially methylated CpG sites according to the distance to the TSS and CpG island.**

|  |  | **Island** | **N_Shore** | **S_Shore** | **N_Shelf** | **S_Shelf** | **OpenSea** |
| --- | --- | --- | --- | --- | --- | --- | --- |
| Up-regulated | Intergenic | 2849 | 675 | 412 | 63 | 57 | 312 |
|  | 3'UTR | 129 | 52 | 20 | 4 | 3 | 36 |
|  | Body | 2809 | 652 | 368 | 90 | 76 | 446 |
|  | 1stExon | 770 | 49 | 21 | 0 | 2 | 25 |
|  | 5'UTR | 2010 | 300 | 193 | 31 | 24 | 167 |
|  | TSS1500 | 2012 | 588 | 386 | 19 | 5 | 83 |
|  | TSS200 | 2155 | 297 | 149 | 10 | 9 | 146 |
| Down_regulated | Intergenic | 146 | 390 | 236 | 425 | 454 | 4358 |
|  | 3'UTR | 14 | 38 | 38 | 32 | 19 | 459 |
|  | Body | 181 | 335 | 237 | 269 | 282 | 3283 |
|  | 1stExon | 12 | 16 | 12 | 0 | 7 | 249 |
|  | 5'UTR | 18 | 89 | 67 | 55 | 67 | 741 |
|  | TSS1500 | 15 | 235 | 206 | 44 | 34 | 1033 |
|  | TSS200 | 7 | 30 | 22 | 11 | 17 | 606 |

**Table S5: Genome-wide cis-regulation between DNA methylation and gene expression according to the distance to the TSS.**

|  | TSS200 | TSS1500 | 5'UTR | 1stExon | Body | 3'UTR |
| --- | --- | --- | --- | --- | --- | --- |
| Negative | 2685(4.29%) | 2927(3.78%) | 2727(5.51%) | 771(7.13%) | 4402(2.93%) | 290(1.89%) |
| Positive | 281(0.45%) | 577(0.75%) | 739(1.49%) | 62(0.57%) | 5010(3.34%) | 542(3.52%) |
|  |  |  |  |  |  |  |

**Table S6: Genome-wide cis-regulation between DNA methylation and gene expression according to the distance to the CpG island.**

|  | Island | N_Shelf | N_Shore | OpenSea | S_Shelf | S_Shore |
| --- | --- | --- | --- | --- | --- | --- |
| Negative | 5112(3.40%) | 472(1.90%) | 2434(3.87%) | 3361(1.90%) | 406(1.82%) | 2017(4.10%) |
| Positive | 1157(0.77%) | 475(1.91%) | 1001(1.59%) | 3538(2.01%) | 380(1.70%) | 660(1.34%) |
|  |  |  |  |  |  |  |

**Table S7:** **Comparison of the performance of different methylation markers for classifying colon cancer and normal tissues.**

| **CpG site^a^** | ***LIFR*-related sepcific  biomarkers** | ***SEPT9*-related  biomarkers** | **4 (of 8) biomarkers  reported by Hao et. al.^b^** | **cg26256223  reported by Naumov et. al.^c^** | **cg16993043  reported by Naumov et. al.^c^** | **cg07990546  reported by Naumov et. al.^c^** |
| --- | --- | --- | --- | --- | --- | --- |
| **TCGA** | 0.963(0.947,0.000) | 0.954(0.892,0.053) | 0.999(0.974,0.000) | 0.997(0.947,0.000) | 0.983(0.974,0.000) | 0.990(0.921,0.000) |
| **GSE48684** | 0.864(0.828,0.000) | 0.926(0.844,0.050) | 0.978(0.922,0.024) | 0.995(1.000,0.049) | 0.966(0.906,0.024) | 0.954(0.938,0.000) |
| **GSE53051** | 0.938(0.857,0.000) | 0.935(0.800,0.000) | 0.963(0.971,0.056) | 0.992(0.971,0.056) | 0.979(0.971,0.111) | 0.930(0.886,0.056) |
| **GSE42752** | 0.963(0.955,0.049) | 0.906(0.818,0.024) | 0.970(0.909,0.000) | 1.000(1.000,0.000) | 0.996(1.000,0.049) | 0.998(1.000,0.024) |
| **GSE77718** | 0.954(0.948,0.062) | 0.971(0.917,0.062) | NA | 0.994(0.990,0.052) | NA | 0.973(0.969,0.062) |
| **GSE77955** | 0.972(0.846,0.000) | 0.902(0.817,0.000) | 0.993 (0.923,0.000) | 0.930(0.923,0.091) | 0.937(0.923,0.091) | 0.993(0.923,0.000) |
|  |  |  |  |  |  |  |

^a^AUC of ROC curve analyses (true positive rate (sensitivity), and false-positive rate (1 - specificity)) are given in this table.

^b^Hao et al. reported 8 CpG sites as colon cancer diagnostic biomarkers. Four sites were utilized to construct a diagnostic model after eliminating the influence of multicollinearity generated from several sites.

^c^Naumov et al. reported 14 CpG sites as colon cancer diagnostic biomarkers. Only one site could be utilized to construct a diagnostic model at a time after eliminating the influence of multicollinearity generated from several sites and three sites were randomly selected to build the logistic regression model.
